# Biomechanics of circulating cellular and subcellular bioparticles: beyond separation

**DOI:** 10.1186/s12964-024-01707-6

**Published:** 2024-06-17

**Authors:** Behrouz Aghajanloo, Hanieh Hadady, Fatemeh Ejeian, David W. Inglis, Michael Pycraft Hughes, Alireza Fadaei Tehrani, Mohammad Hossein Nasr-Esfahani

**Affiliations:** 1 Department of Mechanical Engineering, Isfahan University of Technology, Isfahan, Iran; 2 Department of Animal Biotechnology, Cell Science Research Center, Royan Institute for Biotechnology, ACECR, Isfahan, Iran; 3 Department of Science, Research and Technology (DISAT), Politecnico di Torino, Turin, Italy; 4 School of Engineering, Faculty of Science and Engineering, Macquarie University, Sydney, NSW 2109 Australia; 5 Department of Biomedical Engineering, Khalifa University, Abu Dhabi, United Arab Emirates

**Keywords:** Physical/Mechanical properties, Biomarkers, Circulating cells, Extracellular vesicles

## Abstract

Biomechanical attributes have emerged as novel markers, providing a reliable means to characterize cellular and subcellular fractions. Numerous studies have identified correlations between these factors and patients’ medical status. However, the absence of a thorough overview impedes their applicability in contemporary state-of-the-art therapeutic strategies. In this context, we provide a comprehensive analysis of the dimensions, configuration, rigidity, density, and electrical characteristics of normal and abnormal circulating cells. Subsequently, the discussion broadens to encompass subcellular bioparticles, such as extracellular vesicles (EVs) enriched either from blood cells or other tissues. Notably, cell sizes vary significantly, from 2 μm for platelets to 25 μm for circulating tumor cells (CTCs), enabling the development of size-based separation techniques, such as microfiltration, for specific diagnostic and therapeutic applications. Although cellular density is relatively constant among different circulating bioparticles, it allows for reliable density gradient centrifugation to isolate cells without altering their native state. Additionally, variations in EV surface charges (-6.3 to -45 mV) offer opportunities for electrophoretic and electrostatic separation methods. The distinctive mechanical properties of abnormal cells, compared to their normal counterparts, present an exceptional opportunity for diverse medical and biotechnological approaches. This review also aims to provide a holistic view of the current understanding of popular techniques in this domain that transcend conventional boundaries, focusing on early harvesting of malignant cells from body fluids, designing effective therapeutic options, cell targeting, and resonating with tissue and genetic engineering principles.

**Graphical Abstract:**

This review provides a comprehensive and clear overview of the size/shape, stiffness, density, and electrical properties of circulating cellular/noncellular

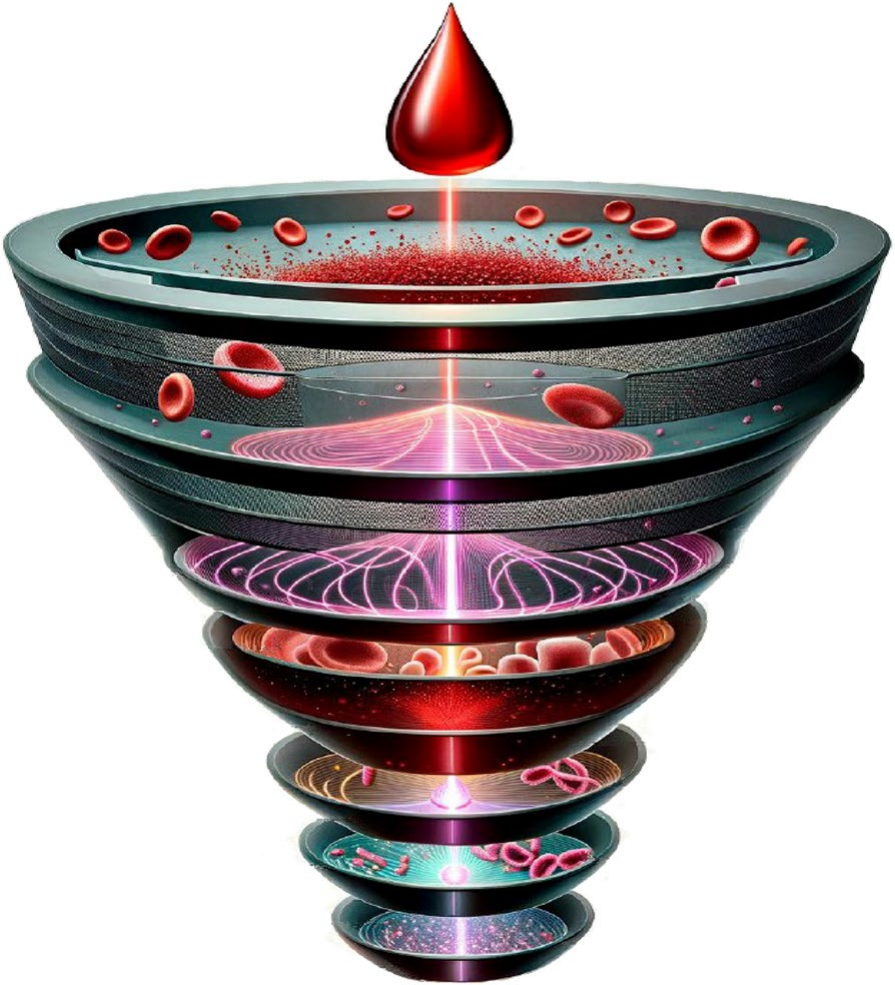

## Introduction

Circulating bioparticles, including cells and extracellular vesicles released into the blood, have been the focus of researchers in academic and clinical applications at the onset of various investigations [1–5]. These bioparticles are found in different liquid or semiliquid environments within the body, such as in the lymph, saliva, and blood. They are popular candidates for experiments due to their specific adaptations to their environment, which differentiates them from their adherent counterparts [6, 7]. For example, as they normally exist in a dispersed and suspended state, they are ideal candidates for cell separation and sorting techniques, whether traditional or emerging. These methods can be classified based on their inherent properties and can be divided into two major groups: antibody-based and label-free methods. The first group is generally based on the immunological properties of cells. In particular, specific surface antigens facilitates label/affinity-based cell separation using techniques such as fluorescence or magnetic-activated cell sorting (FACS or MACS, respectively), ensuring high separation purity and specificity [8, 9] providing high separation purity and specificity. However, these methods are costly and necessitate extensive sample preparation and reagent consumption. In contrast, label-free methods, whether active methods like dielectrophoresis and acoustophoresis [10, 11] or passive methods like inertial separation and centrifugation [12–14], exploit the inherent physical, mechanical, and electrical properties of particles for separation. These techniques are typically simpler, quicker, and more cost-effective, making them ideal candidates for point-of-care diagnostics and mitigating biases from antigen affinity and expression levels. Meanwhile, the most common method involves using density gradients to separate blood into red and white blood cells and platelets. However, ongoing research has introduced label-free techniques that prioritize simplicity, reliability [15], detection accuracy, clinical relevance, reduced invasiveness [16], and the potential for process automation [17]. These methods consider physical and mechanical properties such as size, stiffness, shape, morphology, and electrical characteristics. The goal is to avoid biases seen in methods that depend on the affinity of antigens and their varying expression levels [18–20].

The physical and mechanical properties of bioparticles can serve as distinctive markers, offering insight into their overall health and functional status. Specifically, irregularities in circulating cells—such as pathological changes in deformability and density—have been linked to various diseases [21, 22]. This potential suggests a promising avenue for establishing versatile label-free detection techniques, with potential applications in diverse approaches for separation, analysis, and diagnosis [4]. Label-free techniques are versatile and have been used in various studies ranging from research to clinical applications. They play a crucial role in point-of-care (POC) diagnostics, isolating rare cells, biosensing, and the development of rapid cell-based analysis kits [23].

Although many studies have investigated the physical/mechanical properties of bioparticles, the increasing development of label-free methods requires more comprehensive reviews to cover all related information together. This concept helps develop tools for personalized medicine owing to the reasonable variation between cells of the same type in different people (patient-specific) and even between cells of a specific type of a single person’s body (precision medicine). Furthermore, the physical/mechanical characteristics of circulating bioparticles are valuable for understanding the cellular pathophysiology of several ailments and diseases resulting from cell abnormalities. This concept could provide a critical path toward the use of different therapeutic strategies, such as cell therapy, immunotherapy, targeted drug delivery, and gene therapy, in precision medicine [24–27]. Notably, achieving suitable performance heavily relies on comprehensive knowledge of the exploitable physical/mechanical properties of bioparticles, which are significantly different. As these characteristics are fundamentally of various types, classifying them would be helpful for providing an easy-to-follow dataset of the physical/mechanical properties of bioparticles.

Historically, notable variations in the density, deformability, morphology, and size of bioparticles have been widely applied for designing different label-free sorting techniques, such as density gradient centrifugation [24, 25], filtration [26, 27], and inertial focusing [28]. This approach has long been used for sperm preparation processes in assisted reproduction to separate sperm from bacteria, germ cells, WBCs, and other components present in semen [29, 30]. The second group employs differences in the electric and dielectric properties of cells, such as the conductivity and permittivity of the cytoplasm and membrane, as the basis of a sorting mechanism [31–33]. Integrating emerging technologies such as acoustophoresis, dielectrophoresis, deterministic lateral displacement (DLD), and inertial microfluidics opens up new possibilities for simplifying cell sorting strategies while maintaining high cell viability and preserving the natural state of cells [34].

This review describes a comparative study of the physical and mechanical properties of circulating bioparticles and discusses the methods used for the characterization and measurement of these properties. Additionally, a brief survey of the strengths and limitations of label-free platforms for commercialization has been conducted.

### Classification of circulating particles

**Fig. 1:**
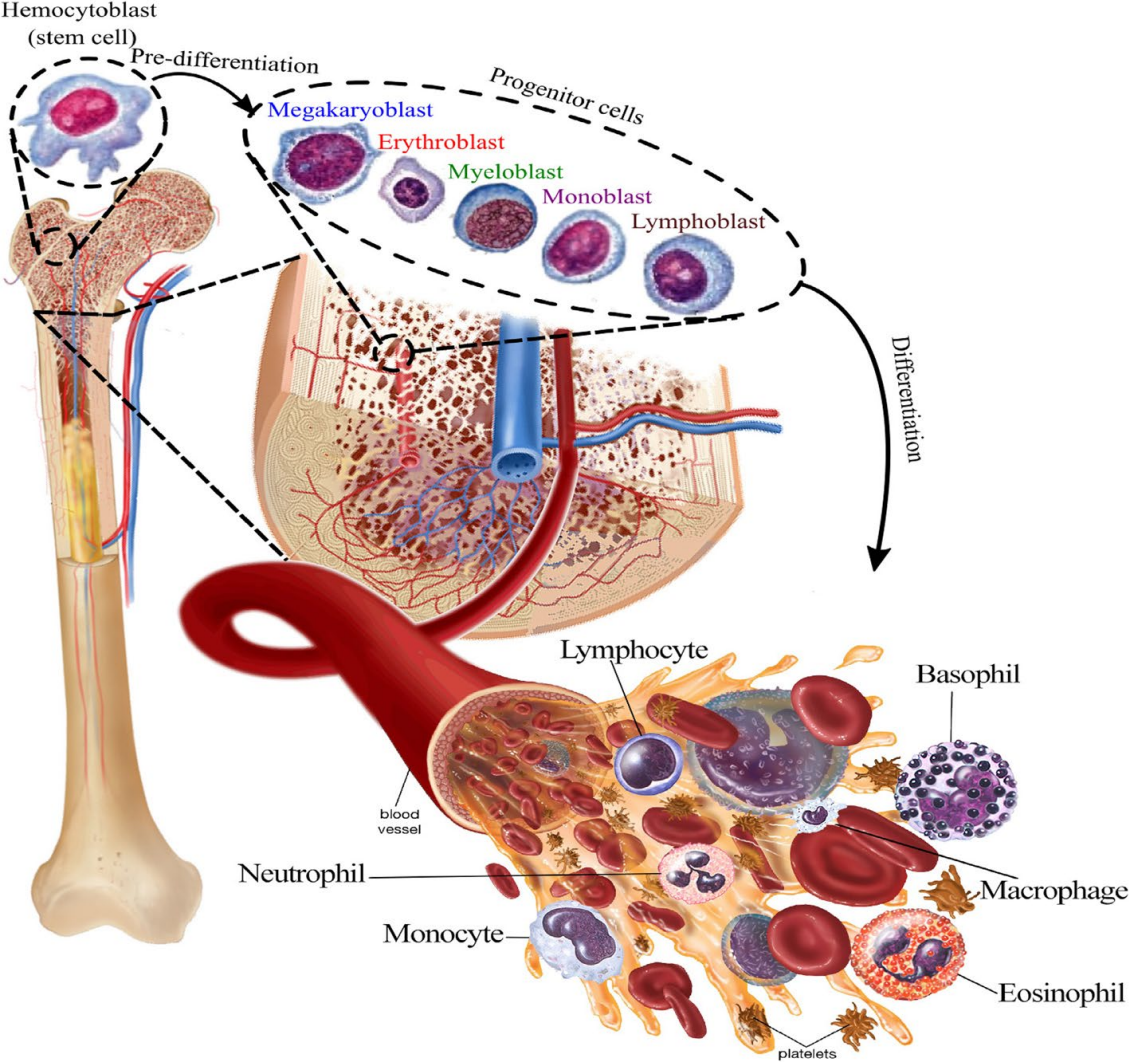
Blood cell journey from the bone marrow to the vessels. Blood cells develop from hematopoietic stem cells that predominantly reside in the bone marrow microenvironment. Hematopoiesis is accompanied by the release of mature WBCs, RBCs, and platelets into the lymph and blood vessels

Circulating cells, which naturally do not require an attachment surface, are present in body suspensions and are typically found in fluids such as blood or lymph [35]. Blood has always been the principal biofluid in the circulating cell separation arena because the blood circulatory system carries a variety of cell suspensions, including its constituent cells—namely, red blood cells (RBCs), white blood cells (WBCs, also called leukocytes), and platelets—as well as various pathological cells and rare mesenchymal and hematopoietic stem cells released from the bone marrow (Fig. 1). Notably, the isolation of mesenchymal stromal cells (MSCs) is of significant clinical interest due to their potential role in regenerative medicine and tissue engineering. These cells, which can differentiate into a variety of cell types, are valuable for treating conditions such as osteoarthritis, myocardial infarction, and spinal cord injuries [34, 36, 37].

Additionally, trace amounts of cancerous tumor-derived cells, so-called circulating tumor cells (CTCs), can be identified in blood samples, which is considered the ‘leukemic phase’ of solid cancers [38, 39]. Circulating fetal cells (CFCs) are a distinct type of cells that enter maternal circulation as early as 4–6 weeks of pregnancy. These cells are classified into three subpopulations: fetal leukocytes, nucleated red blood cells (nRBCs), and trophoblasts. Their importance is due to their potential for early diagnosis of infant disorders by noninvasive acquisition from maternal blood [31–33, 40–42]. This wide range of cells has been used in diverse fields of study, including hematology, oncology, and immunology, in addition to their application in some modern fields, such as regenerative medicine, targeted drug delivery, evaluation of drug efficacy, and tissue engineering [7]. Circulating cells are classified from different perspectives, such as their origin, function, motility, shape and morphology, and surface markers. These factors will be discussed in the following sections [1].

### Normal circulating cells

Blood cells are derived from a common ancestral cell type (i.e., haematopoietic stem cells (HSCs)) in the bone marrow and are suspended in plasma; this process is called haematopoiesis. During hematopoiesis, two principal precursor cells, referred to as myeloid stem cells and lymphoid stem cells, are produced [43]. Myeloid stem cells differentiate into all classes of myeloid cells, including (i) erythrocytes; (ii) thrombocytes, which form platelets; (iii) myeloblast lineage cells, which form granulocytes; (iv) monoblasts, which form promonocytes; and (v) monocytes [44]. On the other hand, lymphoid progenitor cells give rise to all types of lymphocytes, including T cells, B cells, and natural killer (NK) cells, which are released into the blood circulation and play distinct roles in immunity [45].

RBCs are biconcave disc-shaped cells that navigate across the body, exchanging oxygen and carbon dioxide between the lungs and organs and tissues. WBCs are part of the body’s immune system and play a pivotal role in the body’s defense against infections and foreign pathogens. Based on the presence of visible granules in the cytoplasm of WBCs, these cells are divided into two major groups: granulocytes and agranulocytes. These cells generally have a multilobular nucleus called polymorphonuclear leukocytes (PMNs). Neutrophils, eosinophils, and basophils are three types of granulocytes [45].

Platelets, or thrombocytes, are small, nonnucleated, and colorless cell pieces produced by megakaryocyte (MK) fragmentation in the bone marrow. They contain all the cytoplasmic compartments of MKs, including granules, mitochondria, translational apparatus, and mRNAs. Megakaryocytes are routinely generated from myeloid progenitor cells during the megakaryocytopoiesis process, through which megakaryoblasts sequentially differentiate into promegakaryocytes and megakaryocytes. Finally, thrombocytes are released from mature MKs in the blood circulation and play a prominent role in blood coagulation [46].

Circulating fetal cells are scarce, with an abundance of 2–6 cells per milliliter of maternal blood during the second trimester of pregnancy [47]. Among them, circulating trophoblasts (CTBs) are more interesting due to the expression of unique markers and different physical properties compared to those of typical maternal cells. CTBs play a crucial role in successful pregnancy and are responsible for feeding the embryo. They are initially differentiated from the fertilized egg during the early stages of implantation and cover the placental villi surface during development. Three subpopulations of CTBs have been characterized: cytotrophoblasts (CTs), extravillous cytotrophoblasts (EVTs), and syncytiotrophoblasts (STs) [48, 49].

### Abnormal circulating cells

**Fig. 2:**
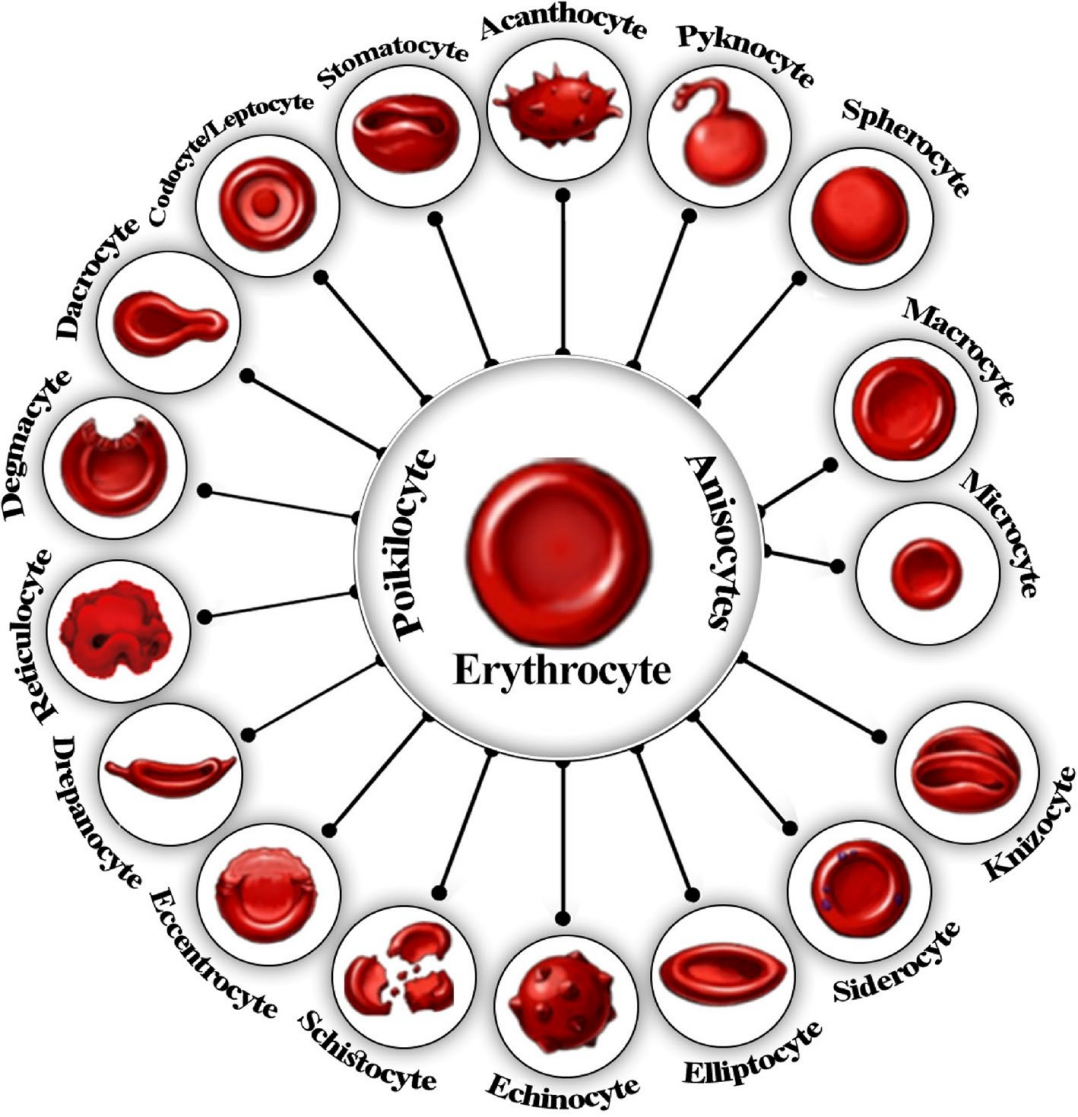
Schematic depiction of red blood cell disorders. A wide range of pathological disorders are associated with abnormalities in the size (anisocytes) or shape (poikilocytes) of erythrocytes, leading to RBC dysfunction

In blood disorders, the frequency, characteristics (including physical and mechanical properties), and function of normal blood cells are altered; their precursor cells, which are usually present in bone marrow, reproduce excessively and find their way to the circulation, leading to disorders such as leukemia (disorders of leukocytes) [50], thalassemia [51], and sickle cell anemia [52]. Epidemiological data show that the prevalence of inherited blood disorders such as sickle cell disorders (SCDs) in individuals between 0 and 15 years of age has considerably increased during the last 20 years [52]. Fig. 2 presents a schematic illustration of the most prevalent disorders impacting the morphological features of RBCs.

CTCs are cancer cells that detach from solid tumors and enter the bloodstream, facilitating tumor metastasis. Therefore, their detection has biomedical significance for determining metastasis prognosis and screening for effective treatments. However, due to the much lower abundance of CTCs (typically 1–20 cells per ml of whole blood, depending on the cancer stage) compared to that of other blood cells, their detection and separation processes face several challenges [53, 54]. Data derived from cancer patients suggest that a mild risk of malignancy (stage I) is usually associated with a CTC count of less than three CTC/ml (0.1–2.9 CTC/ml), moderate malignant potential (stage II and III) with a count of 3–20 CTC/ml, and a high risk of malignancy (stage IV), including metastasis, recurrence, and cancer progression, with > 20 CTC/ml [55].

### Subcellular circulating particles

Subcellular circulating particles are released by various cell types under physiological and pathological conditions [56, 57]. These particles, mainly identified as circulating extracellular vesicles (EVs), represent a heterogeneous population with diverse sizes, membrane compositions, and cytoplasmic components that transfer a specific set of nucleic acids, proteins, and lipids during intracellular cross-talk [58]. Circulating EVs are considered to retain the “signature” of their parent cells, as different agonists and stimuli lead to the formation of a distinct population of vesicles with unique cargo and membrane compositions. EV biogenesis involves the formation of multivesicular bodies followed by either fusion with (intracellular endocytic trafficking pathway) or fission of (outwards budding) the plasma membrane [59]. Subcellular circulating particles are of particular interest as a liquid biopsy tool because of their ability to detect microRNAs (miRNAs) and proteins in EVs, which are now widely studied before and after therapy to achieve individualized treatment [60]. According to the MISEV2018 guidelines [61], EV subtypes are classified according to their size, density, biochemical composition, and parent cell properties instead of using terms such as exosomes, microvesicles (MVs), and apoptotic bodies. EVs classified by size are categorized as either “small EVs” (sEVs) less than 200 nm in diameter or “medium/large EVs” (m/lEVs) with a diameter larger than 200 nm. EV density is not generally a helpful classification criterion, with density occupying a narrow range between 1.1 and 1.19 g/ml [62–64]. Considering their biochemical composition, EVs are composed of diverse integral membrane tetraspanins (e.g., CD81, CD9, and CD63) [65], scaffold proteins (e.g., ERM proteins, syntenin, Alix), and proteins of the ESCRT machinery [66, 67]. The condition of the parent cells is also considered an essential factor in EV composition. For example, apoptotic bodies or small apoptotic vesicles (~ 100-5,000 nm) are released when cells undergo apoptosis [64]. However, the study of EVs is associated with several technical challenges, including purification, quantification, and isolation, which must be addressed before EV-based approaches can be widely adopted. Isolating EVs is accompanied by multiple complications due to their nanoscale size and heterogeneity, which increases the risk of co-isolating contaminants with similar sizes, such as cellular debris, protein complexes, and lipoproteins, when using methods solely based on size, like size exclusion chromatography [68, 69]. On the other hand, traditional methods for EV enrichment, like ultracentrifugation, are time-consuming and complex. In recent years, novel microfluidic approaches have emerged, offering a combination of innovations, particularly for personalized medicine and precise biomarker detection [70]. Considering all these challenges, there is no single method for isolating EVs, and the best approach may involve combining different methods, which consequently affects the molecular content and biological activity of the isolated EVs [71, 72]. Recent developments in sequencing, omics, imaging, and nanoparticle technologies have paved the way for the precise prompt detection of low-concentration EVs in blood plasma. We have attempted to review and discuss the properties and characterization methods of EVs in later sections to advance this approach.

In addition to circulating EVs, blood plasma contains biomolecules such as DNA, RNA, and proteins derived from tumorous or healthy tissues [73, 74]. However, the challenges in their measurement include their low specificity and instability, and these limitations have hindered their application in liquid biopsy [75].

### Physical properties of circulating cells

Circulating cells can be investigated based on their physical and mechanical properties, such as shape and morphology, and their qualitative properties, including size, density, stiffness, and electrical properties. In blood cell disorders, circulating cell properties can vary in size (anisocytosis), shape (poikilocytosis), color, and the presence of inclusion bodies (Fig. 2) [76, 77].

Of particular importance to the cell separation procedure is the utilization of the physical and mechanical properties of the target cells, providing a valuable tool for manipulating these cells. To choose an appropriate method, the intrinsic properties of the target cells to be sorted must be well recognized. However, this property on its own cannot be trusted for an efficient and precise isolation and/or manipulation approach [78, 79]. Cell size varies widely among different types of cells. Conversely, the variation in cell density is generally much lower than that in size and volume, while density is a helpful criterion for separating WBCs and RBCs. In the RBC population, there is only 0.5% variation in density [79], almost 20 times less than the extent of variation in cell size [80]. Hence, it could be considered a more reliable basis of separation [79–81]. Another separation criterion is stiffness, which measures the resistance of a cell to deformation under an applied force and determines the deformability of the circulating cells when passing through narrow capillaries [82–84].

### Size and morphology

Although cell size is usually estimated based on diameter, surface area, and volume, cell length is generally considered for manipulation purposes [78]. Cell diameter significantly influences cell adhesion to the endothelium [79] during metastasis [80] or recruitment to a site of tissue injury [81]. Assuming that cells are approximately spherical, only one of the three parameters above would need to be measured, from which the other two can be calculated. For instance, optical and Coulter techniques measure cell diameter and volume, respectively. Therefore, the surface area and volume for optical procedures remain to be calculated, while the diameter and surface area need to be estimated for the Coulter technique [82].

The results of cell size measurements must be applied with caution. Potential mistakes in interpreting results include *(i)* sample preparation steps, such as spreading and flattening due to heaviness during 2D size measurements using microscopy; *(ii)* centrifugation, fixation, and staining of the cells; and *(iii)* inherent factors, such as culture media components, cell viability status, and cell cycle stages [83]. Some of the frequently used methods to study cell size include differential interference contrast (DIC) microscopy, bright-field microscopy, flow cytometry, counterpetting, and blood smears [1, 83].

Circulating cells with separable size ranges are exploitable for sorting, although the reported size range overlaps can sometimes be challenging. They include epithelial tumor cells and circulating trophoblasts (15–25 μm in diameter), red blood cells (erythrocytes are 6–8 μm, and fetal nucleated red blood cells (fNRBCs) are 9–12 μm biconcave disks) and peripheral blood lymphocytes (7–10 μm in diameter) [83–86]. Table 1 illustrates the size range of various circulating bioparticles, including their approximate count and measurement techniques. An efficient separation method requires a distinctive size difference among cells to be identified. Hydrodynamic and gravitational forces play significant roles in particle relocation in modern separator platforms such as microfluidic devices, a well-known technology used for handling liquids on the order of micro/nanoliters. Cells of different sizes experience unequal inertial and drag forces (hydrodynamic force phenomena), resulting in different cell trajectories during their travel. The larger the size is, the larger the force [6]. Therefore, cell size significantly determines cell fate in a microfluidics channel.

**Table 1:** The size (diameter, area, volume) of cellular and subcellular circulating objects

Circulating objects type		Size	Measuring method	Approximate count	Ref.
Normal circulating cells	Platelet	7–13 µm^3^	Dual optical tweezers stretching technique	150–400(x10^9/L)	[87]
		2–3 μm	Blood smear	200–400(x10^9/L)	[88]
		10.5 ± 0.5 µm^3^	DIC microscope	NA	[89]
	Erythrocyte	84.59 µm^3^	Quantitative absorption imaging	NA	[90]
		80–99 µm^3^	Dual optical tweezers stretching technique	4.40–5.80(x10^12/L)	[87]
		100.6 ± 4 µm^3^	DIC microscope	NA	[89]
		7–8.5 μm	Blood smear	3.8–7 (x10^12/L)	[88]
Granulocyte	Neutrophils	9–16 μm	Blood smear	60–62% in blood	[91–94]
		15 μm	Blood smear	2.6–7(x10^9/L)	[88]
	Basophils	10–16 μm	Blood smear	0.4-1% in blood	[91–94]
		10–14 μm	Blood smear	0.0–0.1(x10^9/L)	[88]
	Eosinophil	9–16 μm	Blood smear	2.3-3% in blood	[75–78]
		14 μm	Blood smear	0.05–0.4(x10^9/L)	[88]
Agranulocytes	Lymphocyte	Small: 7–8 μm Large: 12–18 μm	Blood smear	30% in blood	[75–78]
	Monocyte	12–20 μm	Blood smear	5.3-6% in blood	[75–78]
		15–20 μm	Blood smear	0.8–0.8 (x10^9/L)	[88]
Abnormal circulating cells	BC- CTCs^a^	120.4 µm^2^	CELLSEARCH® CTC Test	CTC count (including clusters) depends on the cancer progression, whereby: • < 3 CTC/ml (0.1–2.9 CTC/ml) is correlated with Stage I of cancer progression • 3–20 CTC/ml is correlated with Stages II and III of cancer progression • > 20 CTC/ml is correlated with Stage IV of cancer progression	[95]
		851.6 ± 45.8 µm^c^	DIC microscope		[89]
		290 ± 200 µm^2^	CELLSEARCH® CTC Test		[96]
		13.1 μm	Microscope		[97]
		29.8 ± 6.5 μm	Microscope		[98]
		33.9 ± 8.3 μm	CELLSEARCH® CTC Test		[98]
		32.0 ± 5.8 μm	CELLSEARCH® CTC Test		[98]
	PC – CTCs^b^	83.6 µm^2^	CELLSEARCH® CTC Test		[79]
		180 ± 145 µm^2^	CELLSEARCH® CTC Test		[96]
		10.7 μm	Microscope		[96]
	CRC – CTCs^c^	44.6 µm^2^	CELLSEARCH® CTC Test		[79]
		186 ± 153 µm^2^	CELLSEARCH® CTC Test		[96]
		11 μm	Microscope		[97]
	BLC – CTCs^d^	57.8 µm^2^	CELLSEARCH® CTC Test		[79]
EVs^g^	Small EVs	< 200 nm < 100 nm	• NTA^e^ • Flu-SEC^f^ • Electron microscopy • Flow cytometry	1334/µl in RBC unit 64% of small EVs	
	Medium/ large EVs	> 200 nm		7.6 ± 3.2–11.2 ± 18.5(x10^9/mL) in serum	[99–103]

^a﻿^Breast cancer patient-derived CTCs

^b﻿^Prostate cancer patient-derived CTCs

^c﻿^Colorectal cancer patient-derived CTCs

^d﻿^Bladder cancer patient-derived CTCs

^e﻿^NTA: Nanoparticle tracking analysis

^f﻿^Flu-SEC: Size exclusion chromatography with on-line fluorescence detection

^g﻿^Extracellular vesicles

The physical properties of CTCs have been well described previously by Hao et al. [83]. In general, the size of CTCs is typically in the range of 4–30 μm, with a higher nucleus-to-cytoplasm ratio than that of WBCs [5]. CTCs are highly heterogeneous, depending on their physiological conditions, origin, and patient-by-patient status [104]. For instance, it is evident that CTCs isolated from central venous blood have a larger average area (77.59 µm^2^) than those harvested from peripheral venous blood (62.28 µm^2^) [104–106]. Not surprisingly, the tissue origin of CTCs also significantly impacts these characteristics. In particular, Coumans et al. reported that breast cancer CTCs have a total volume of 851.6 ± 45.8 μm [89], as measured by microscopy. Moreover, ovarian cancer CTCs have a total volume of 518.3 ± 24.5 µm^3^, as measured by DIC microscopy [107]. According to microscopy reports, prostate cancer CTCs have a diameter of 10.7 μm [106], while the diameter of melanoma CTCs ranges from 9 to 19 μm [108].

**Fig. 3:**
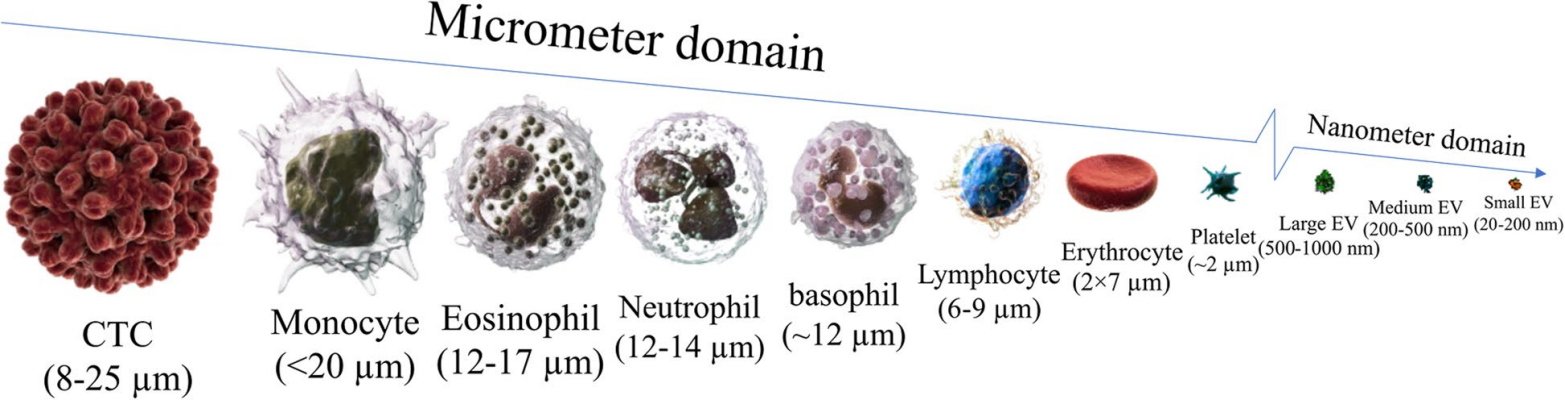
Various morphological features of circulating bioparticles. Normal blood cells are 2–20 micrometres long with different levels of circularity and granularity, while noncellular particles appear in nanometric order with less structural complexity

Circulating cells may exhibit distinct morphological characteristics, which can be used as an exploitable separable parameter along with size differences (Fig. 3). For example, CTCs have been shown to have a rounder shape than leukocytes [96]. An exciting study by Wu et al. showed that the morphological characteristics of CTCs can predict the prognosis of lung cancer patients, as small and irregularly shaped nuclei are correlated with an increased risk of disease recurrence [109]. Although Jaferzadeh et al. reported that erythrocytes preserved their normal morphology during rapid temperature elevation (less than a one-hour time course from 17 °C to 41 °C), they found that some of their profile features, such as projected surface area and sphericity coefficient, may be altered [110]. Additionally, White et al. have conducted a comprehensive study that explains how the discoid form of platelets can be affected by temperature variation. They reported the loss of platelet disc shape by chilling from 37 °C to 4 °C, which was reversed by rewarming to 37 °C [111]. Readers are encouraged to refer to Bain [112] for a comprehensive guide to normal and abnormal blood cell types and morphologies.

### Stiffness

**Fig. 4:**
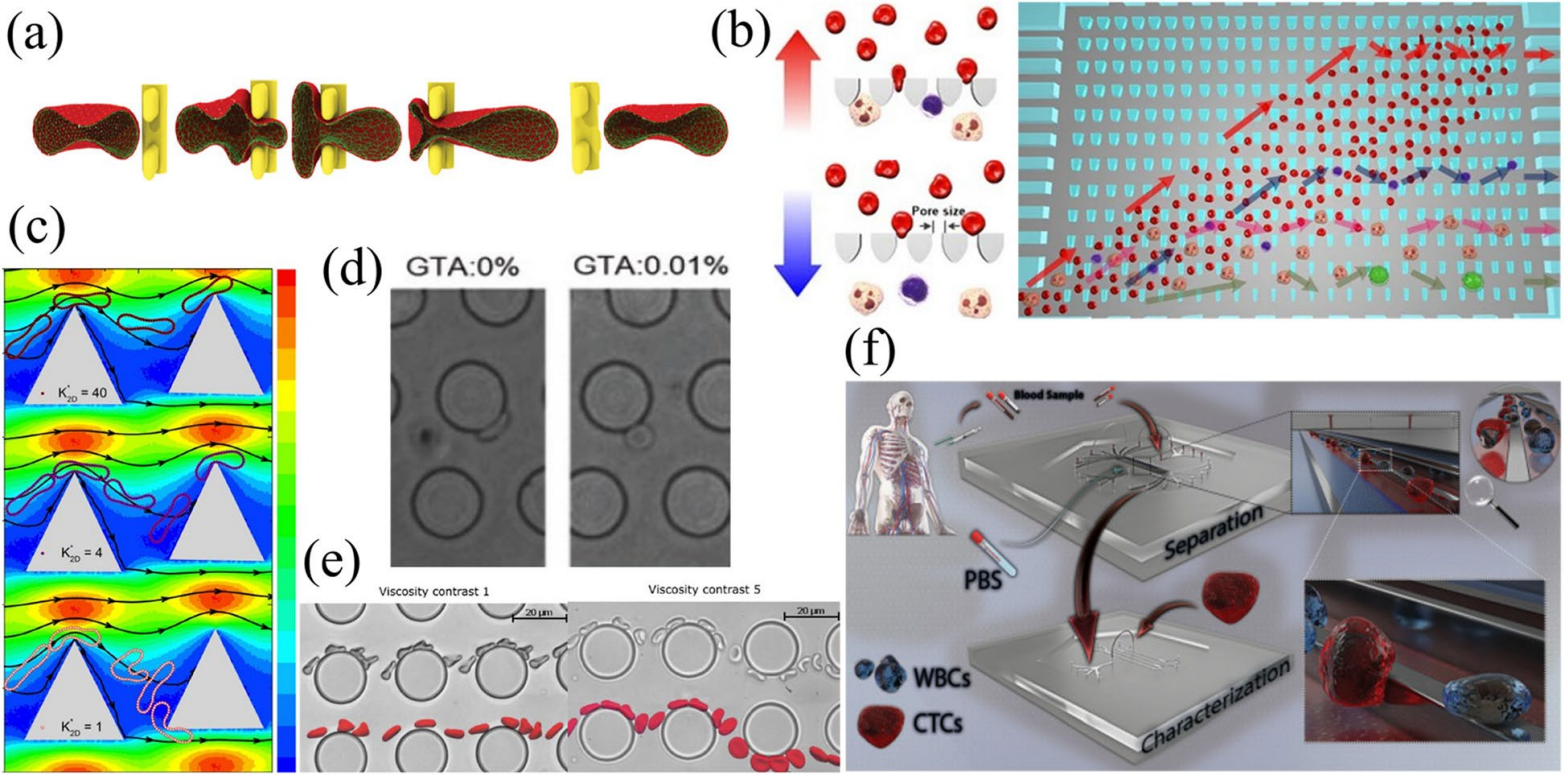
The stiffness/deformability of circulating cells is an exploitable feature for separation. **a** Simulation of passing normal RBCs through interendothelial slit (IES)-like features [145]; (**b**) direct isolation of leukocytes from whole blood using microfluidic ratchets [146]; (**c**) simulation results for deformation facing RBCs interacting with DLD triangular posts having different rigidities [122]; (**d**) interaction of glutaraldehyde-treated (stiff) and nontreated (compliant) RBCs with DLD cylindrical posts [119]; (**e**) sorting of RBCs based on their dynamical properties [147]; and (**f**) deformability-based CTC isolation from whole blood based on a slanted weir [148]. Figures reproduced with permission from references

Stiffness, defined as the resistance of a cell to deformation under an applied force [113], describes the ability of the circulating cells to deform if needed [7, 114]. Deformability determines the potential of cells in the circulatory system to travel through the microvasculature (Table 2). It is considered to be one of the fundamental characteristics of circulating cells and can change under pathological conditions [115]. Therefore, deviations in normal cell deformability may serve as a proxy for diagnosis and prognosis. The bulk separation of circulating cells is commonly achieved by sedimentation-based methods that use the physical properties of the cells, such as size and density. However, deformability often plays a significant role in the enrichment efficiency of retention-based methods (Fig. 4(a)). It is often assumed that both the size and density of the cells determine their ability to be enriched or to pass through the pores of the filter. Larger and denser bioparticles are more likely to be retained. For example, sedimentation-based filtration retains denser cells, and microfiltration techniques trap larger particles while allowing smaller ones to pass through [24]. However, depending on the applied driving force and deformability of the target cells, circulating cells (which are compressible and deformable) can be squeezed through pores smaller than the original cell size [116]. Therefore, in filtration-based approaches, the resistance of cells to deformation is more critical than cell size for cells of similar sizes (Fig. 4(b)). For example, the largest leukocytes (neutrophils and monocytes with 8.13–8.62 μm cell diameters measured by flow cytometry and Coulter counter techniques) can be separated from the smallest ones (lymphocytes and platelets with 6.08–7.04 μm cell diameters measured by flow cytometry and Coulter counter techniques) based on size differences. The greater retention rate of monocytes compared to that of neutrophils is attributed to their greater stiffness (0.15 and 0.055 mdyn/µm, respectively), as measured by the cell poker technique [108]. Deformability can also be used in separation methods, mostly known as size-based techniques, such as DLD [117–125]. Accordingly, considerable investigations are dedicated to discovering the interactions of circulating cells in different manners with various DLD posts. Several numerical, experimental, and comparative studies (Fig. 4(c)-(e)) have been conducted to determine how deformability affects the tracking of circulating cells. With the evolution of deformability-based separation methods, corresponding microfluidic platforms are being upgraded to increase the throughput, relying on direct isolation of circulating cells from whole blood (Fig. 4(f)). In addition to DLD, other microfluidic platforms have been developed for deformability-based cell sorting, including conical-shaped microfilters [126], inertial microfluidics [127], microfluidic gradual filters [128], and inertial-based spiral microchannels [129]. For instance, spiral microchannels with various loops were designed and assessed to separate deformable CTCs using an arbitrary Lagrangian–Eulerian (ALE) and finite element method (FEM) approach. The results revealed that the trajectory of CTCs was affected by cell deformability, cell size, number of loops, and channel depth when crossing through the spiral channel [129].

**Table 2:** Stiffness of cellular and subcellular circulating objects

Circulating objects type		Stiffness	Measuring method	Ref.
Normal circulating cells	Effective stiffness^a^ of fresh RBCs	26.5 ± 8.3 µNm^−1^	AFM	[130]
	Effective stiffness of 6 weeks stored RBCs	95 ± 7 µNm^−1^	AFM	[130]
		108 ± 18 µNm^−1^	Microfluidics	[130]
	Deformability index^b^ of RBCs	0.0698 ± 0.024	Dual optical tweezers stretching technique	[87]
	Elongation index of RBCs Neutrophils	0.0618 ± 0.024	AFM	[131]
		0.156 ± 0.087 kPa	AFM	[132]
	E^c^ of RBCs	7.57 ± 3.25 kPa	AFM	[133]
	E of WBCs	1.99 ± 1.84 kPa	AFM	[134]
		1.6 × 10^4^ kPa	Microfluidics	[135]
	E of WBCs	1.962 ± 0.517 kPa	AFM	[136]
Abnormal circulating cells	ALL^d^ patients with leukocytosis symptoms	1·1 kPa	AFM	[137]
	ALL from asymptomatic patients	0·06 kPa	AFM	[121]
	E of Ovarian cancer HEYA8 line	0.494 ± 0.222 kPa	AFM	[138]
	E of Ovarian cancer HEY line	0.884 ± 0.529 kPa	AFM	[138]
	MDA-MB-231^e^	0.2062 ± 0.241 kPa	Microfluidic	[139]
	Malignant urothelial cells	0.1964 ± 0.0424 kPa	Micropipette aspiration	[140]
	E of melanoma cells	0.876 ± 0.127 kPa	AFM	[136]
	Average E of the lung, breast, & Pancreatic tumor cells	0.53 ± 0.10 kPa	AFM	[141]
EVs	Bending modulus of RBC-derived EVs	15 ± 1 k_B_T	AFM (modified Canham-Helfrich Nano indentation model)	[142]
	E of Exosomes	1.45–8.16 × 10^5^ kPa	AFM (Hertz-contact indentation model)	[143]
	E of large EVs (90–120 nm)	2.6 − 7.3 × 10^4^ kPa	AFM (Hertz-contact indentation model)	[143]
	E of small EVs (60–80 nm)	0.7– 4.20 × 10^5^ kPa	AFM (Hertz-contact indentation model)	[143]^112^
	E of human malignant metastatic bladder cell-derived EVs	2.8 × 10^5^ kPa	AFM (Thin Shell indentation model)	[144]

^a﻿^Calculated as $\text{k} = \frac{{\text{F}}_{\text{s}}}{\text{L}}$, where ${\text{F}}_{\text{s}}$ is the shear force acting on the end of the cell, and $\text{L}$ is the cell deflection [130]

^b﻿^Calculated as $$\text{D}\text{e}\text{f}\text{o}\text{r}\text{m}\text{a}\text{b}\text{i}\text{l}\text{i}\text{t}\text{y} \ \text{i}\text{n}\text{d}\text{e}\text{x}= \frac{ \text{F}\text{i}\text{n}\text{a}\text{l} \ \text{s}\text{t}\text{r}\text{e}\text{t}\text{c}\text{h}\text{e}\text{d} \ \text{l}\text{e}\text{n}\text{g}\text{t}\text{h} \ \text{o}\text{f} \ \text{R}\text{B}\text{C}- \text{I}\text{n}\text{i}\text{t}\text{i}\text{a}\text{l} \ \text{l}\text{e}\text{n}\text{g}\text{t}\text{h} \ \text{o}\text{f} \text{R}\text{B}\text{C}}{\text{I}\text{n}\text{i}\text{t}\text{i}\text{a}\text{l} \ \text{l}\text{e}\text{n}\text{g}\text{t}\text{h} \ \text{o}\text{f} \ \text{R}\text{B}\text{C}}$$[87]

^c﻿^E: Elastic (Young’s) modulus

^d﻿^ALL: Acute Lymphoblastic Leukaemia

^e﻿^Mean stiffness of Human breast metastatic cancer

Because RBCs need to pass through narrow capillaries for gas transport, deformation is necessary for the optimal performance of RBCs [7]. The biconcave shape of RBCs facilitates their deformability, which is in turn affected by cell sphericity (surface at a given volume), the mean cell haemoglobin concentration (internal viscosity), and membrane dynamics [149]. Renoux et al. reported that changes in these factors influence RBC isotonic deformability (elongation in the direction of the flow) under shear stresses above three pascals. Their increase is associated with a decrease in deformability. However, below this threshold, only the loss of membrane elasticity could cause a reduction in RBC deformability [150].

A reduction in deformability occurs due to RBC aging, resulting in an increase in hemoglobin levels and a reduction in membrane elasticity [151]. This decrease can also be observed in RBC pathogenesis, including sickle cell disease, spherocytosis thalassemia, and immune hemolytic anemia. Compared with RBCs, leukocytes have greater stiffness, are less deformable, and can cause capillary obstruction. It has been reported that microfilament organization (i.e., f-actin), stimulated by chemotactic factors, contributes to changes in neutrophil stiffness and subsequent neutrophil sequestration in pulmonary capillaries [152]. The role of the actin network in the elastic response of a cell has also been described for other cell types [153]. Deformability appears to increase during the differentiation process, as reported for the HL-60 cell line [154]. Similarly, expelling the nucleus towards the end of the differentiation process results in softer and smaller RBCs, as measured by real-time deformability cytometry (RT-DC) and AFM [155].

CTCs are significantly stiffer than RBCs and WBCs because they originate from nonhematological tissues such as epithelial cells [7]. Therefore, leukocytes are more motile than circulating cancer cells when measured during the moving state [156]. During activation, leukocytes such as neutrophils [157] and lymphocytes [158] can undergo dramatic morphological changes from a semirigid spherical state to a flattened and highly deformable state [7]. However, in most cancers, including lung, breast, prostate, skin, ovarian, and oral cancers, cancerous cells have lower stiffness than normal cells, which is much more significant in metastatic cells that facilitate migration and invasion [138, 159, 160]. It has been shown that in the same sample, metastatic cancer cells are approximately 73 ± 11% less stiff than benign mesothelial cells [141].

Changes in cytoskeletal structure were observed in pathological erythrocytes, resulting in two to three times greater cell stiffness than that of normal cells. In contrast to most cancer cells, leukemic cells, including ALL, AML, CML, and CLL cells, exhibit increased stiffness compared to that of normal blood cells [30, 157, 161]. This increase in cell stiffness and Young’s modulus is attributed to changes in the structure of spectrin, a molecular anomaly in hemoglobin structure, or impaired ATP metabolism [153]. Additionally, it has been reported that increased metastatic capacity of human cancer cells can lead to reduced cell stiffness. In this sense, metastatic cells derived from the pleural fluid of patients with lung, breast, and pancreatic cancer were approximately 70% softer than benign cells [162]. Moreover, the varying amount and content of proteins within tumor cell-derived EVs might influence the mechanical properties of similar-sized vesicles. Due to the presence of membrane proteins, natural vesicles demonstrate greater stiffness than liposomes [163].

The cell stiffness can be described as the Young’s modulus or elastic modulus, which is obtained by fitting the curve of force versus indentation. The force applied by the cantilever in the AFM method is determined by multiplying the deflection by the spring constant of the cantilever following Hooke’s law [164]. The units of Young’s modulus are pounds per square inch (psi) in the English system and newtons per square meter (N/m^2^) or Pascal in the metric system. Phenomenological parameters, including the deformability index (DI) and elongation index (EI), depend on the flow velocity. However, the effective stiffness (calculated as the shear force over the cell length), which reflects the inherent mechanical properties of circulating cells, is mainly independent of the flow velocity under low velocities (< 0.03 m /s) [130]. Therefore, the effective stiffness is a more reliable parameter for describing the stiffness of circulating cells (specifically RBCs) than the DI or EI is.

Several methods can be used to characterize cell stiffness, including micropipette aspiration, magnetic bead rheometry, magnetic and optical tweezers, particle-tracking microrheology, and atomic force microscopy (AFM) [87, 161, 165, 166]. AFM, which calculates Young’s modulus, has been widely used for determining the elastic and viscoelastic properties of whole cells and offers advantages such as controlled operation force, minimum imposed damage, and ease of sample preparation compared to other techniques [164, 167]. Current AFM methods are suitable for identifying adherent cells but cannot be used for determining the stiffness of suspension cells due to the slipping of cells under load. Rosenbluth MJ et al. proposed a method for assessing the stiffness of suspension cells whereby cells are immobilized by pipetting in microfabricated wells and subsequently subjected to the force exerted by the tip of a cantilever [132]. AFM also allows simultaneous visualization of important cellular structures such as the cytoskeleton and is often combined with microscopy approaches [153].

**Fig. 5:**
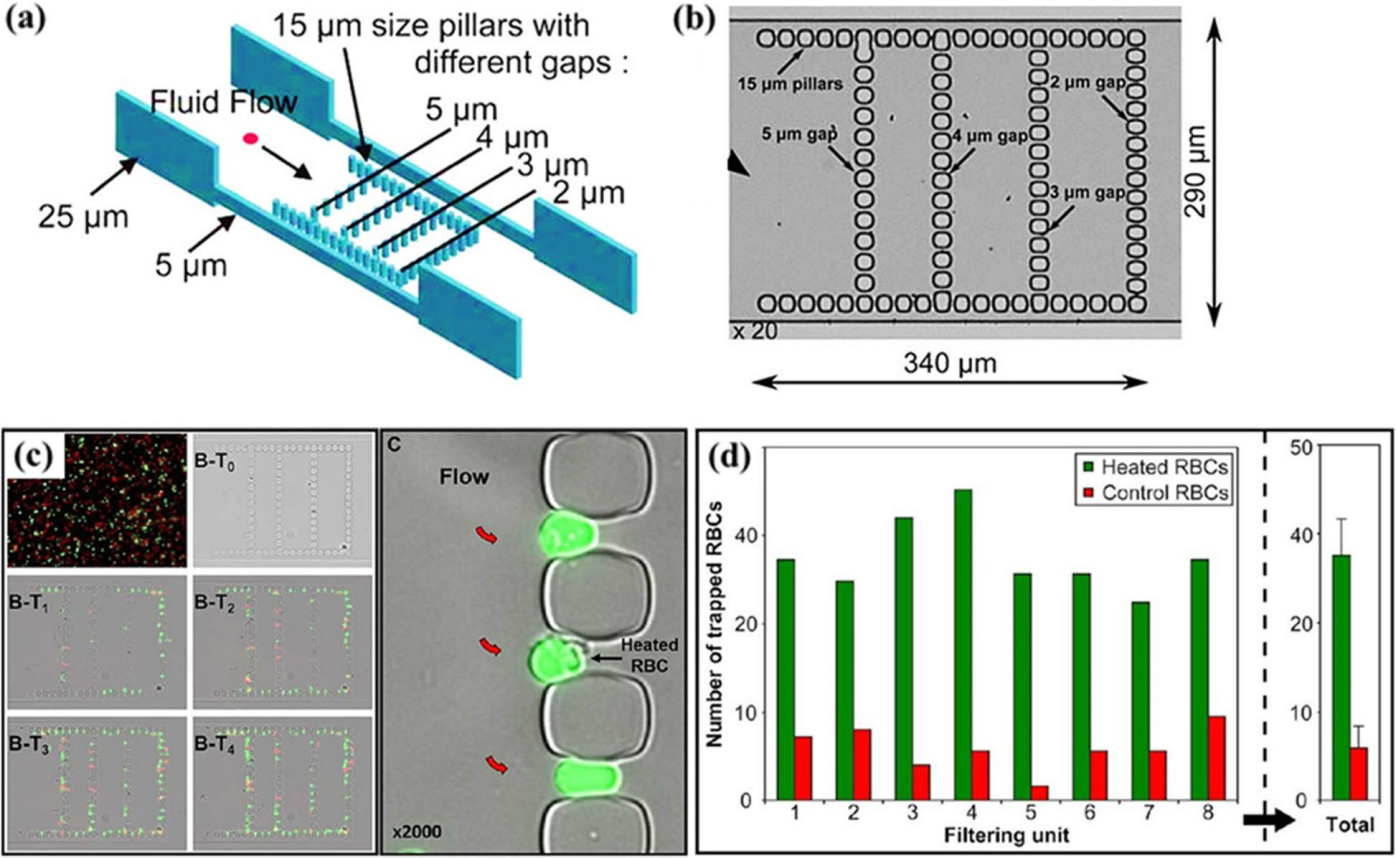
Effect of cell physicomechanical properties on deformability. **a** Design and (**b**) manufacturing process of a spleen-like chip. Each filtering unit included fifty-three 2-µm-wide slits between the 15-mm pillars that generated the boundaries of the lattice. **c** Increasing accumulation of poorly deformable heated RBCs in slits 4 (T1), 8 (T2), 12 (T3), and 16 (T4) minutes after initiation of RBC perfusion through the filtering unit (flow is from left to right). Ellipsoid or quasispherical aspect of heated RBCs retained in 2 mm wide slits (flow is from left to right, red arrows). **d** Number of poorly deformable heated RBCs (green) and normal RBCs (red) retained in the narrow slits of each of the eight filtering units in a chip and the mean values (right panel) [172]. Figures reproduced with permission from references

Notably, the deformability of cells is affected by other physicomechanical properties [168]. For instance, RBCs exposed to 50 °C for 15 min exhibited increased rigidity, leading to decreased size, spheroid shape, and increased irreversible stiffness [169] (Fig. 5). As shown in Fig. 5 (a and b), stiffly heated (green) and normal RBCs (red) were circulated within the chambers of a spleen-like microfluidic device, and their retention was followed for up to 16 min (Fig. 5c). The data revealed that compared with control RBCs, stiffly heated RBCs were poorly deformable, and the number of these RBCs retained in the slits, particularly the 2-µm slits, was significantly greater (approximately seven times greater) during perfusion. As another example, CTCs derived from prostate cancer patients were isolated from whole blood using a microfiltration system based on their size. According to the obtained data, CTCs exhibit greater elasticity and membrane smoothness than nontumor cells, which indicates their potential invasiveness and mobility in the peripheral circulation [170]. Smoothness represents cell mobility, the distribution of surface proteins, and the loss of cell polarity [171]. Consequently, the high deformability and smoothness of CTCs can be linked to morphological changes in these cells to mesenchymal-like cells during the epithelial–mesenchymal transition (EMT) process for malignant invasion [170].

To investigate the relationship between cell shape and elastic behavior, Ravetto et al. developed a microfluidic system capable of measuring the deformation of activated (lipopolysaccharide-treated) and nonactivated monocytes [173]. In this regard, compared with no treatment, lipopolysaccharide treatment led to cytoskeleton reorganization and increased the elastic compressive modulus of monocytes by up to 340%. However, the shear modulus and stiffness of the treated cells decreased (up to 88%) in contrast to those of the nontreated cells.

### Density

Density is routinely defined as the mass-to-volume ratio of a cell and varies according to different health statuses and growth phases. As shown in Table 3, the density variation between circulating bioparticles was less than that between cell sizes [174]. Notably, cell aging in RBCs leads to an increase in density due to an increase in the mean corpuscular hemoglobin concentration [151]. This process may accelerate in RBC disorders, including hereditary spherocytosis, as RBC dehydration occurs [175]. Conversely, the density of leukocytes and ovarian cancer-associated high-definition CTCs (HD-CTCs) is reportedly approximately 3.5–4.5 times less than that of RBCs [107].

The spatially variable density of the cells results in their semitransparent appearance under standard bright field microscopy. This fact contributes to significant phase lags of the transmitted waves and the utilization of phase contrast microscopy [176]. Correspondingly, noninterferometric quantitative phase microscopy (NI-QPM) and differential interference contrast (DIC) microscopy were used to measure the cellular dry mass and total volume, respectively [107]. Although density gradient centrifugation [177] is a well-known method for measuring the average density of a cell population, measuring the mass and density of a single cell suspended in a fluid can be complicated. Several techniques have been proposed over the past few years by researchers. For example, Zhao et al. presented an optically induced electrokinetic (OEK) platform that rapidly determines the density and mass of a single leukemic cell by combining sedimentation theory, computer vision, and microparticle manipulation techniques [75].

The density gradient centrifugation technique is widely used to enrich circulating trophoblasts from maternal blood. Kliman et al. reported that the density of CTBs ranged from 1.048 to 1.062 g/ml, [178] which was confirmed by further studies with some minor differences in trophoblast subpopulations [179, 180]. Moreover, acoustophoresis has recently been highlighted as an advanced microfluidic technology that separates particles and cells based on ultrasonic waves. This technique applies a half-wavelength ultrasound standing wave field throughout a microchannel to control the particles’ mobility in the liquid. Consequently, acoustophoresis allows for the separation of cells across a wide range of sizes, densities, and stiffnesses with gentle handling, simply by applying acoustic excitation [181]. So far, this method has been employed to separate various cells and particles, including CTCs [182], blood cells [183], and extracellular vesicles [184]. In addition, Franziska Olm et al. reported that they successfully separated bone marrow-derived mesenchymal stromal cells from other cellular populations using microchip acoustophoresis [34]. The data revealed that the separated cells demonstrated approximately a 20% higher proliferation rate and a 1.7-fold increase in clonogenic potential compared to the input sample. Furthermore, these cells were significantly smaller in size, with a mean diameter of 14.5 ± 0.4 μm, compared to the center outlet fraction, which had a mean diameter of 17.1 ± 0.6 μm.

**Table 3:** Density of cellular and subcellular circulating objects

Circulating objects type		Density (kg/m^3)^	Measuring method	Ref.
Normal circulating cells	MD^a^ of monocytes	1067–1077	Ficoll-Hypaque density gradient centrifugation	[185]
	MD^a^ of lymphocytes	1073–1077		
		1070		
	RBCs	> 1077	Ficoll-Hypaque density gradient centrifugation	[186]
	Platelets	1040	Density gradient centrifugation	[187]
	WBC	850 ± 4	Optical quantification combining NI-QPM^b^ and QDIC^c ^microscopy	[107]
	Basophil	1072–1078	Density centrifugation on Percoll	[188]
	Neutrophil	1080–1090	Ficoll-Hypaque density gradient centrifugation	[189]
	Eosinophil	1090–1100		[189]
Abnormal circulating cells	Ovarian cancer-associated HD-CTCs	650 ± 6	Optical quantification of the dry mass density	[190]
	DRBCs^d^	> 1120	Phthalate density-distribution profile method	[191]
	HL-60^e^	1045–1095	Combining “sedimentation” principle with OEK^f^	[192]
EVs	Vesicles ranging from 30 to 150 nm	1130–1190	DGU^g^	[61–63]
	Apoptotic bodies (500–4000 nm)	1160–1280		

^a﻿^Median cell density

^b﻿^Noninterferometric quantitative phase microscopy technique

^c﻿^Quantitative differential interference contrast microscopy

^d﻿^Erythrocyte dehydration associated with sickle cell disease

^e﻿^Human leukemic cells

^f﻿^Optically induced electrokinetic

^g﻿^Density gradient ultracentrifugation

### Electrical properties

The zeta potential of mammalian cells is affected by their net surface charge under different physiological/environmental conditions (Table 4) [1, 193, 194]. Moreover, when placed in an electric field, single-cell behavior is dictated primarily by its dielectric properties (i.e., membrane and cytoplasm conductivity and permittivity). It is subsequently governed by two main mechanisms: conductive polarization (the physical movement of the free charges) and dielectric polarization (the field-induced disturbance of bound charges). Depending on the frequency domain of the imposed electric field, one of the mentioned mechanisms becomes dominant. The membrane acts as a shield to the applied electric field at low frequencies. As the frequency increases, dielectric polarization becomes more significant due to its faster response time. The polarizability of the cells to the surrounding medium (physiological conditions) determines the next dipole moment acting on the cells [195, 196].

**Table 4:** Electrical properties of cellular and subcellular circulating objects

Circulating objects type		Electrical properties	Measuring method	Ref.
Normal circulating cells	Zeta potential of RBCs	Minimum = − 9.3 mV Maximum = − 15 mV	Double optical tweezers	[197]
	Conductivity of RBCs membrane	5 × 10 –5 S/m	Analytical measurements	[198]
	Conductivity of RBCs cytoplasm	0.5 S/m	Analytical measurements	[198]
	Membrane capacitance of RBCs	10.89 mF/m^2^	Dielectrophoresis	[199]
	Mean specific membrane capacitance of monocytes	15.3 ± 4.3 mF/m^2^	ROT	[200]
	Conductivity of monocytes cytoplasm	0.56 ± 0.10 S/m		
	Cytoplasm permittivity of monocytes	126.8 ± 35.2		
	Mean specific membrane capacitance of T-lymphocytes	10.5 ± 3.1 mF/m^2^		
	Conductivity of T-lymphocytes cytoplasm	0.65 ± 0.15 S/m		
	Cytoplasm permittivity of T-lymphocytes	103.9 ± 24.5		
	Mean specific membrane capacitance B-lymphocytes	12.6 ± 3.5 mF/m^2^		
	Conductivity of B-lymphocytes cytoplasm	0.73 ± 0.18 S/m		
	Cytoplasm permittivity B-lymphocytes	154.4 ± 39.9		
	Mean specific membrane capacitance of granulocytes	11.0 ± 3.2 mF/m^2^		
	Conductivity of granulocytes cytoplasm	0.60 ± 0.13 S/m		
	Cytoplasm permittivity of granulocytes	150.9 ± 39.3		
Abnormal circulating cells	Membrane capacitance of murine ovarian cancer cells	Early stage: 15.39 ± 1.54 mF/m^2^ Late stage: 26.42 ± 1.22 mF/m^2^	Dielectrophoresis	[201]
	Membrane capacitance of MCF-10A^a^	19.4 ± 1.4 mF/m^2^	Whole-Cell Impedance Spectroscopy	[202]
	Membrane capacitance of MCF-7^a^	18.6 ± 1.1 mF/m^2^		
	Membrane capacitance of MDA-MB-231^a^	16.3 ± 1.7 mF/m^2^		
	Membrane capacitance of MDA-MB-435^a^	15.7 ± 1.2 mF/m^2^		
	Membrane capacitance of A549^b^	16.95 ± 2.93 mF/m^2^	ROT	[203]
	Cytoplasm conductivity of A549^b^	0.23 ± 0.05 S/m		
	Cytoplasm permittivity of A549^b^	100		
EVs	Surface conductance of BxPC-3 and AsPC-1 derived EVs^c^	6-12.5 nS	Conductance-Based measurements	[204]

^a^Breast cancer cell line

^b^Lung cancer cell line

^c^Pancreatic tumor cells

Any kind of disease or abnormality might cause a change in the electrical traits of cells. As reported, the dielectric permittivity of cancerous RBCs, which is associated with the thickness of the hydrated shell around the aberrant RBC membrane, increases, independent of the cancer type [205]. Similarly, the unit membrane capacitance of tumor cells is greater than that of leukocytes [1]. The membrane conductance and cytoplasmic conductivity of erythrocytes regulate cellular electrophysiology, including circadian rhythms, which depend on the cycling of cytoplasmic K + levels and exhibit temperature-compensated behavior [206, 207]. However, cellular self-regulation and likely homeostasis maintain the relatively stable dielectric properties of erythrocytes [208]. This fact reflects the importance of these parameters as promising candidates for diagnostic purposes [209]. In addition to conventional techniques, including electrorotation (ROT) [210–212], impedance spectroscopy [202], and dielectrophoresis (DEP) [199], custom-designed microfluidic methods have been used to measure the dielectric properties of cells even at single-cell resolution [213, 214]. Among circulating cells of similar size, monocytes and T lymphocytes exhibit the greatest (15.3 ± 4.3 mF/m^2^) and smallest (10.5 ± 3.1 mF/m^2^) membrane capacitance, respectively, as measured by the ROT [200, 215]. In addition, DEP revealed that the membrane capacitance and cytoplasm conductivity of mouse ovarian surface epithelial (MOSE) cells increased from 15.39 ± 1.54 mF/m^2^ for the early malignancy stage to 26.42 ± 1.22 mF/m^2^ for high-grade cancerous MOSEs [201, 216]. RBCs were reported to have a membrane capacitance of 10.89 mF/m^2^, as measured by dielectrophoresis [199]. The membrane potential and zeta potential can be altered to approximately − 20 to 20 mV by exposing the membrane to different media, as extensively investigated by Hughes M et al. [217]. These findings demonstrate the potential of exploiting the electrical properties of circulation cells for separation and diagnosis purposes [218].

The electrical and morphological characteristics of the BeWo cell line, an established model of human trophoblast cells, were investigated by Ramos et al. [219]. The conductance of the cells increased by 122% (from 0.72 to 1.60 nS/cell) in response to exposure to cyclic adenosine monophosphate (cAMP) for less than 15 min. This cell type also exhibited a maximum permeability of approximately 70 Ω ⋅ cm^2^ [220].

### Physical properties of circulating EVs

Regardless of the secretion mechanisms (endosome-origin “exosomes” or plasma membrane-derived “ectosomes” (microparticles/microvesicles)), no consensus has yet been reached on specific biomarkers of different EV subtypes because of overlapping physical characteristics [59, 64]. EVs exhibit various sizes, shapes, and densities that make it challenging to categorize them based on a single factor. These properties are affected primarily by the expression of a specific protein or lipid [58], the action of various pathways (e.g., metabolic pathways) [221], and characterization or isolation techniques [222]. Tables 1, 2, 3 and 4 show the different sizes, stiffnesses, densities, and electrical properties of the EV subtypes.

Purification of a particular subtype of EVs is difficult, and coisolation of a heterogeneous population may occur due to the size, content, function, and source of EVs. As a consequence, the effective classification of EV subtypes is based on combinations of two or three parameters, such as size, density, and origin. Correspondingly, isolation must occur by combining several methods aimed at different properties (e.g., size or density). Circulatory EVs come from several sources. Platelet-derived EVs are the most abundant EVs in the blood [223] and the first discovered EVs [224]. In addition to platelets, other blood cells [225] and cells that reside in various tissues [226], such as those in the central nervous system[227], as well as cancer cells [228], also release EVs into circulation. The diverse populations of EVs that circulate in the blood contribute to numerous physiological and pathological processes [223].

Several techniques can be employed to characterize the physical properties of EVs. However, each method introduces biases, and sample preparation significantly affects the final results. Arraud et al. discovered three morphologically distinguished EV subpopulations in a healthy platelet-free plasma sample using the cryo-electron microscopy technique [99]. The spherical-shaped EVs were found to be 30 nm–1 μm in diameter, while the number of EVs with a length-to-width ratio greater than five were referred to as tubular EVs (average length of 2.2 ± 1.3 μm). Indeed, vesicles called large fragments (1 to 8 μm) constitute approximately 10% of the EV population. Differences in EV sources, sizing methods, and isolation techniques have contributed to size variability in different studies [59, 222]. Nanoparticle tracking analysis (NTA), dynamic light scattering (DLS), and resistive pulse sensing methods estimate the hydrodynamic sizes of EVs based on their mobility in solution. It is generally believed that this parameter is sensitive to the proteins and glycans that adhere to the EV membrane [229, 230]. The membrane diameter can be precisely measured by transmission electron microscopy (TEM) regardless of the presence of the attached molecules. However, the sample preparation process may lead to deformation and false reports, including fixation and dehydration. Cryo-TEM and atomic force microscopy (AFM) [228] measure the size of the two-dimensional projections of EVs in a hydrated state and can visualize the lipid bilayer and vesicle internal structures. The single-particle interferometric reflectance imaging sensor (SP-IRIS) technique can detect EVs simultaneously in serum or whole blood [231, 232]. Most recently, Wallucks et al. introduced a highly sensitive technique for size photometry (SP) of EVs that relies on interferometric scattering (iSCAT) imaging of immersed extracellular vesicles on a glass coverslip. The integration of the SP method with fluorescence imaging (SPFI) enables the system to proceed through a long process with a yield of more than 10,000 EVs in 7 min. The EV sizing limit ranges from approximately 35 to 200 nm. Moreover, the SP system has been incorporated with flow cytometry for analysing EVs, including deformation monitoring using fluorescent tags [233]. Almost all of these methods revealed that EVs have a spheroidal morphology. While trace amounts of EVs with nonspherical morphology (tubular-like, [99]elongated, etc.) have been reported, they are likely to be the result of contamination with lipoprotein particles [234] or exomers [143], physical-induced fragmentation, and/or biological processes [64, 235]. EVs purified from a single cell type also exhibit diverse morphologies and compositions. A study revealed nine distinct EV morphologies from human mast cell-1 (HMC-1) that perform specific functions and shaping to serve as a cellular communicator. However, the majority of the population exhibited a round-shaped vesicle morphology (81.7% of HMC-1-derived EVs, as measured by cryo-TEM) [236].

The optical and non-optical methods explained earlier, electrical characterization techniques rely on either the electrical detection of a byproduct (e.g., from a redox reaction catalyzed by an enzyme) [237] or the intrinsic charge of the EV [238]. EVs routinely carry a negative surface charge ranging from − 6.3 to -45 mV [204, 239] depending on the suspending medium conditions. [240]. The surface conductance of BxPC-3 and AsPC-1 cell-derived EVs has been reported to be in the 6-12.5 nS range, which is highly similar to that of other cell-derived EVs. [204]. Considering the growing interest in developing emerging techniques capable of label-free detection of EVs, electrical-based methods, which rely on the polarization of EVs, have gained importance. Some studies have employed electrokinetic forces to relocate and trap EVs on microfluidic devices for liquid biopsy [241–244]. EV isolation can be based on differences in the magnitude or direction of the electrokinetic forces that depend on (a) the intrinsic properties of EVs, including size, shape, protein (cargo), and lipid expression, (b) extrinsic parameters, including the characteristics (frequency, magnitude, shape, phase, etc.) of the applied electric field and (c) the conductivity and permittivity of the suspending medium. Therefore, the extrinsic properties must be adjusted to isolate a specific population of EVs effectively.

However, colloidal nanoparticles behave differently under nonuniform electrokinetic forces that lead to deviation from theory and were first observed by Washizu et al. [245] This phenomenon has since been investigated by Ibsen et al. [242], Tayebi et al. [184], Hübner et al. [246], Hughes [247], Hoettges et al. [248], and Pethig [249]. They observed that the electrokinetic force on 10 nm latex beads (colloidal nanoparticles) is unexpectedly large and dominant, which is due to the surface conductance (the conductivity of the electrolyte in the vicinity of the charged interfaces, i.e., the plasma membrane) of the nanoparticles, which is approximately the same size range as that of the particle. EVs, particularly small ones, are constantly trapped at electrodes and exhibit a positive dielectrophoresis response because of their dominant surface conductance [246, 250]. This method provides a promising tool for liquid biopsy, early cancer diagnosis, and personalized therapy.

### Challenges and perspectives

The diversity of biomechanical characteristics of circulating cells has emerged as a new paradigm in biomarker research. Blood cells, circulating tumor cells (CTCs), circulating fetal cells, and circulating stem cells exhibit variations in size, stiffness, and density influenced by their type, age, and abnormality. At a smaller scale, subcellular particles like EVs possess a wide range of characteristics, such as surface charge, based on their distinct origins and secretion mechanisms. Although these properties provide notable advantages compared to conventional biomarkers (e.g., CD antigens or cytoplasmic biomarkers) for developing rapid, facile, and on-site approaches, there are still practical challenges to their widespread usage. The first challenge is ensuring sufficient exploitable order/range of difference regarding the properties of bioparticles, which determines the quality of separation that can be attained. The other concern is the overlap between two or more separating target type properties since all reported/measured characteristic properties are a mean value of a specific population, which is not exact.

**Fig. 6:**
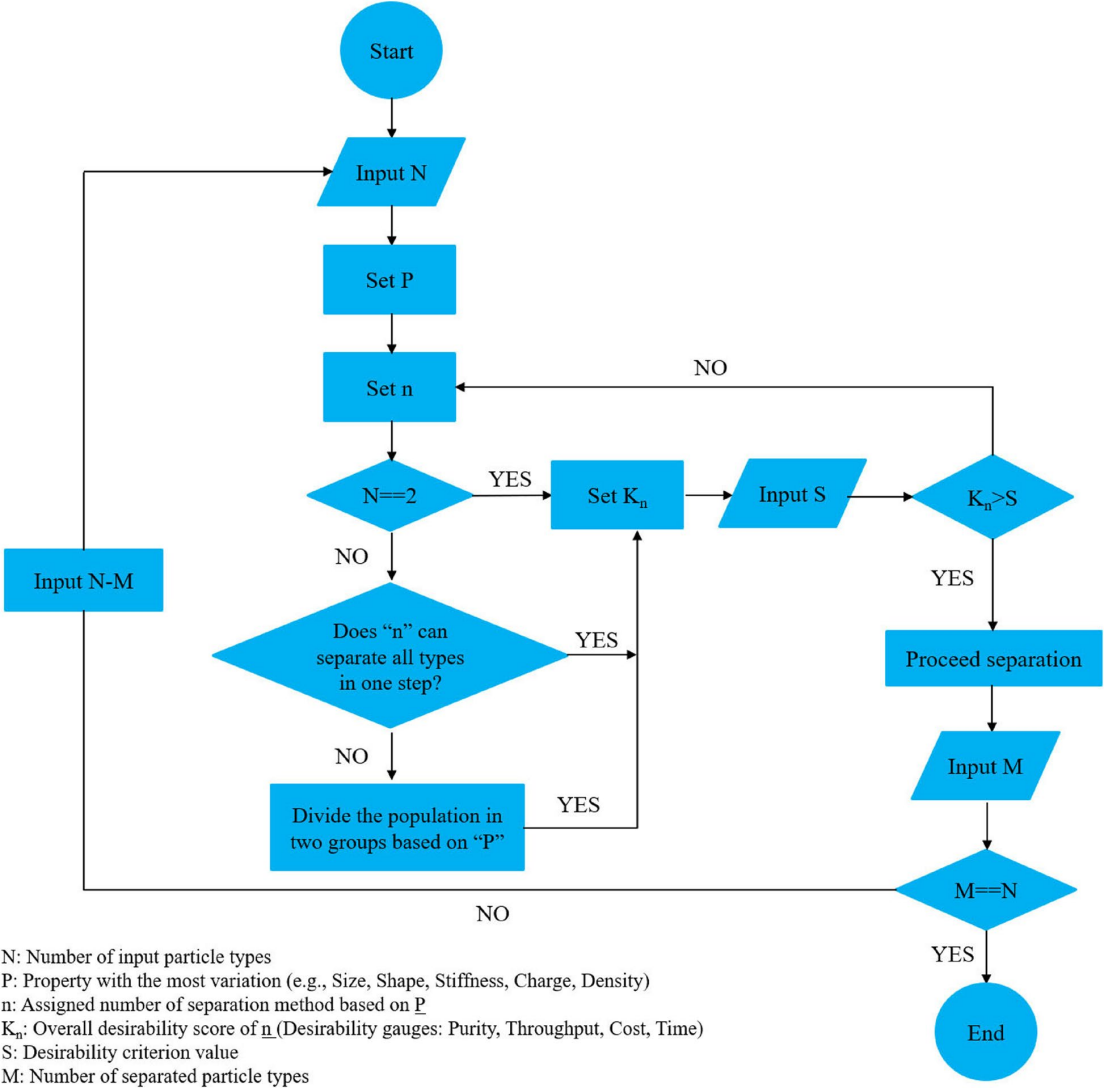
Proposed decision-making flowchart for the particle separation procedure based on physical/mechanical properties. Label-free sorting of a mixed population can be conducted by more than one separation method due to the various physical/mechanical exploitable properties of the particles. To find the best approach to follow, all limitations/potentials and each one’s level of importance should be considered simultaneously. Accordingly, the above flowchart provides simplified/clarified instructions for choosing the best-fitting option

Thus, the first challenge in designing a separation plan is proposing an appropriate technique that relies on the candidate’s property. This approach should meet the requirements while considering the limitations. In this way, the most important factors for selecting the method are the purity and throughput of products, acceptable stress, risk of contamination, operational convenience, and cost and time constraints. Of note, it is impossible to meet all desired requirements and overcome all limitations. Thus, it would be beneficial to allocate the weight of importance to the desirableness factors and assign a score for each technique, considering the overall condition. The number of particle “types” to be sorted is another aspect of consideration regarding the decision-making procedure for choosing the appropriate method for separation. Some mixed populations are composed of different particle types whose difference is not regarding the same property or whose order of difference is not the same. To provide insight, assume, for instance, that the exploitable difference between types A and B is their size, while it is the stiffness between types B and C. It is evident that all three types cannot be separated by the same separation technique or at least in one step. Moreover, variations between particles might stem from the same property but within different ranges (for example, cells versus extracellular vesicles), where one of them could fall outside the working range of the employed method. In some cases, separation techniques can simultaneously separate more than two different particle types [251–256]. However, as mentioned before, if the difference between particle types is due to various properties or different order ranges of the same property, more than one separation technique that can be integrated sequentially [257] or simultaneously [258] in one device is needed. However, when none of the above options are possible, the population should be divided into two distinct groups in the first step. Next, the outputs could be applied as a new sample for completing the process. Accordingly, a convenient decision-making flowchart is proposed to elucidate how to plan for efficient label-free separation considering all aspects (Fig. 6).

The physical/mechanical properties of particular cell types can undergo distinct variations under various environmental conditions. This potential provides a great opportunity over well-known biomarkers to identify an individual cell type in different stages. Moreover, this approach offers an easy-to-exploit and robust tool for detecting and manipulating bioparticles. This emerging area provides new hope for lowering therapy expenses, extensive coverage of disease screening, and improving public health by facilitating diagnosis and prognosis, especially in low-resource countries [231, 232]. However, despite the great potential of label-free techniques, their widespread application is still challenging. This deficiency could be due to different reasons, such as the low product yield of these platforms, difficulty in standardization and scale-up, and incompatibility with conventional analysis tools. Modern techniques for separating bioparticles based on their physical/mechanical properties have been widely applied in preclinical studies; however, they have rarely moved into clinical practice. Nevertheless, progress in high-throughput microfluidic platforms, when combined with artificial intelligence, presents a promising approach capable of providing real-time feedback and optimizing process parameters accordingly. Furthermore, dedicated efforts are being made to standardize separation protocols and establish versatile, applicable guidelines for the consistent application of label-free methods, potentially enhancing their translational potential into clinical settings.

Besides, bridging the gap between research trends and clinical requirements is important. The great variation in the reported parameters is related to the variation in the patients’ backgrounds and disease stages. However, the higher degree of commercialization of label-free methods relies on standardizing measurements and minimizing variability [234, 235]. Some proposed techniques include developing comprehensive databases that cover a wide range of patient backgrounds and disease stages [259] and standardizing protocols for sample collection, preparation, and analysis [260].

As the separation of circulating bioparticles moves towards commercialization, exploiting their properties could allow the implementation of real-time liquid biopsy. Single-cell RNA-Seq platforms, such as 10X Genomics and BD Rhapsody, have revolutionized genomics by enabling precise gene expression analysis at single-cell resolution. These platforms provide intricate insights into cellular heterogeneity and molecular landscapes within complex biological samples [261, 262]. However, these methods come with limitations such as high cost, standardization issues (biases in transcript coverage to detect abundant transcripts), and challenging data analysis requiring computational biology and specialized software [261, 263]. Despite these challenges, they are particularly suitable for uncovering the diversity of RNA transcripts in individual cells, such as studying gene expression profiles, disease mechanisms, and differentiation pathways [264]. In contrast, label-free techniques for circulating bioparticle separation offer a straightforward and unbiased approach that is particularly beneficial for heterogeneous samples because they eliminate the need for complex labelling processes. However, these methods may encounter challenges in accurately identifying specific cell types or capturing detailed molecular information. In addition, nucleic acid-based tests and in vitro diagnostic (IVD) assays, which are available on the FDA platform, provide precise insights into genetic markers crucial for accurate disease diagnosis and prognosis in personalized medicine. Moreover, tools such as CellSearch for CTCs streamline real-time detection of circulating tumor cells but lack tissue- or organ-specific markers for all clinically relevant tumor cells, posing a challenge for comprehensive detection. Striking the right balance between simplicity and molecular resolution is vital when choosing between label-free bioparticle separation and biomarker detection platforms, depending on specific clinical requirements and practical considerations of time and resources [265]. The physical properties of extracellular vesicles can also be used for liquid biopsy and early diagnosis. Currently, a lab-on-a-chip (LOC) platform (Verita™), which uses AC Electrokinetics (ACE) and electrical properties of the EVs for their isolation from whole blood, was developed by Biological Dynamics. The clinical relevance of any emerging technology needs to be eventually determined with patient samples and through large-scale clinical trials.

## Conclusion

This review provides an overview of the existing information and gaps in the size/morphology, stiffness, density, and electrical characteristics of circulating bioparticles for label-free techniques. Three major categories of bioparticles, namely, normal cells, abnormal cells, and subcellular bioparticles, are discussed. Among these properties, size and deformability have attracted the most interest in cellular subgroups because of the reasonable differences between different blood cells, CTCs, and cTBs. However, in the realm of subcellular bioparticles, the focus of studies has centered on their electrical properties. This is attributed to the fact that within the submicron range, collective separation of particles is more feasible and efficient than single-mode separation, a process easily facilitated by an external field. On the other hand, we know a bit about the density of different circulating bioparticles since the exact measurement of this parameter for tiny cells/EVs is still challenging. It is worth noting that the overall stiffness of cells is on the order of KPa, while for cell derivatives (e.g., EVs), it is on the order of MPa. To facilitate the use of these parameters for developing an appropriate separation method, a convenient guide was proposed. Eventually, a combination of two or more separation techniques that could improve the efficiency of the process and address the limitations of each method is highly recommended.

## Data Availability

No datasets were generated or analysed during the current study.
